# Exploring the Efficacy of Hydroxybenzoic Acid Derivatives in Mitigating Jellyfish Toxin-Induced Skin Damage: Insights into Protective and Reparative Mechanisms

**DOI:** 10.3390/md22050205

**Published:** 2024-04-29

**Authors:** Hao Geng, Rongfeng Li, Lichao Teng, Chunlin Yu, Wenjie Wang, Kun Gao, Aoyu Li, Song Liu, Ronge Xing, Huahua Yu, Pengcheng Li

**Affiliations:** 1 CAS and Shandong Province Key Laboratory of Experimental Marine Biology, Center for Ocean Mega-Science, Institute of Oceanology, Chinese Academy of Sciences, Qingdao 266071, China; genghao19@mails.ucas.ac.cn (H.G.);; 2 Laboratory for Marine Drugs and Bioproducts, Pilot National Laboratory for Marine Science and Technology (Qingdao), No. 1 Wenhai Road, Qingdao 266237, China; 3 University of Chinese Academy of Sciences, Beijing 100049, China

**Keywords:** jellyfish, protocatechuic acid, gentisic acid, *Nemopilema nomurai* nematocyst venom, skin necrosis, inflammation

## Abstract

The escalation of jellyfish stings has drawn attention to severe skin reactions, underscoring the necessity for novel treatments. This investigation assesses the potential of hydroxybenzoic acid derivatives, specifically protocatechuic acid (PCA) and gentisic acid (DHB), for alleviating *Nemopilema nomurai* Nematocyst Venom (NnNV)-induced injuries. By employing an in vivo mouse model, the study delves into the therapeutic efficacy of these compounds. Through a combination of ELISA and Western blot analyses, histological examinations, and molecular assays, the study scrutinizes the inflammatory response, assesses skin damage and repair mechanisms, and investigates the compounds’ ability to counteract venom effects. Our findings indicate that PCA and DHB significantly mitigate inflammation by modulating critical cytokines and pathways, altering collagen ratios through topical application, and enhancing VEGF and bFGF levels. Furthermore, both compounds demonstrate potential in neutralizing NnNV toxicity by inhibiting metalloproteinases and phospholipase-A_2_, showcasing the viability of small-molecule compounds in managing toxin-induced injuries.

## 1. Introduction

Presently, a significant global surge in jellyfish populations has been observed, with specific jellyfish species presenting threats within distinct marine ecosystems [1,2]. The unique structure of nematocysts enables jellyfish to rapidly and effectively inject toxins into human skin. As a result, the rise in jellyfish populations has led to a surge in jellyfish stings, endangering the safety of swimmers and waders in warm ocean waters globally [3]. Simultaneously, the growing interconnection between humans and coastal marine areas, driven by technological advancements and globalization, has heightened the risk of jellyfish exposure, intensifying the impact of jellyfish stings [4]. Jellyfish stings in humans result in localized skin reactions, including erythema and scarring. Moreover, systemic symptoms like cardiac and respiratory failure may occur, and contact with certain species can be fatal [5] (e.g., *Chironex fleckeri*, *Physalia physalis*, etc.). The understanding and treatment of symptoms arising from specific cases of human poisoning depend on the bioactive profile of the toxin, which exhibits significant variation across species and geographic distribution [6]. The aforementioned factors contribute to the challenges and complexity involved in treating patients affected by jellyfish stings. The current MSD (Merck & Co., Inc., Rahway, NJ, USA) manual outlines the following treatment options for jellyfish sting poisoning: (I) in situ seawater shower for tentacle removal, (II) baking soda solution shower, (III) application of ice or hot compresses to alleviate pain, and (IV) symptomatic treatment involving nonsteroidal anti-inflammatory drugs (NSAID): hydrocortisone and epinephrine, among others. However, these treatment options are limited, and analyzing the treatment mechanisms for different venomous species is challenging. Consequently, these options often fail to provide immediate relief [7]. Research focusing on developing therapeutic measures following jellyfish stings should prioritize the understanding of the activity exhibited by venom components specific to each species. Moreover, conducting comprehensive preclinical trials is essential to prevent the adoption of ineffective or potentially harmful first aid measures.

*Nemopilema nomurai* (Cnidaria: Scyphozoa) is a globally distributed jellyfish species, known for its large size and high toxicity. It is predominantly found in the marginal seas of eastern Asia, particularly in the Sea of Japan and the East China Sea [8]. In recent years, there have been frequent and increasing population outbreaks of *N. nomurai*, which, coupled with its edibility, have resulted in a significant rise in human encounters, leading to the highest number of severe sting cases within its habitat. *N. nomurai* stings primarily manifest as localized skin symptoms, characterized by intense pain, erythema, urticaria, edema, and occasional fatalities [5,9,10,11]. Recent transcriptomic and proteomic studies have identified the primary component groups in *N. nomurai* venom, including metalloproteases (27.5%), pore-forming toxins (8.7%), phospholipases (7.2%), and neurotoxins (7.2%) [12,13]. Previous studies have demonstrated that these toxic components exhibit various physiological activities, such as hemolysis, cytolysis, proteolysis, and type I hypersensitivity reactions. In vivo experiments have consistently demonstrated that the toxins increase vascular permeability in both the skin and viscera, degrade the cytoplasmic matrix, resulting in substantial edema, skin damage, and muscle injury [14,15]. The potential of metalloproteases (MMPs) and phospholipases A_2_ (PLA_2_s) in assessing sting toxicity as therapeutic targets for jellyfish venom-induced local reactions, and inflammation have been investigated using different enzyme inhibitors, including batimastat, EDTA, and o-phenanthroline [13,16]. Due to the limitations of current treatment options for *N. nomurai* jellyfish stings, there is growing interest among researchers in exploring small-molecule compounds as novel antidotes. These compounds offer the advantages of a low therapeutic threshold and rapid efficacy against local reactions. Small-molecule compounds capable of neutralizing toxins can be classified into two main categories. The first category includes small-molecule enzyme inhibitors, such as batimastat and EDTA, which have shown significant efficacy in reducing the inflammatory response and muscle tissue necrosis induced by *Nemopilema nomurai* Nematocyst Venom (NnNV) [15]. Another category comprises active substances derived from natural products. For instance, the metabolite phenazine-1-carboxylic acid from the fungus *Aspergillus versicolor* SmT07 inhibits the proteolytic and fibrinogenolytic activities of NnNV [17]. Furthermore, epigallocatechin-3-gallate, a natural polyphenol found in green tea, alleviates necrotizing skin lesions and systemic venom intoxication caused by NnNV [18,19]. However, the ongoing preclinical trials concerning small-molecule compounds for stings remain inadequate at present. Concurrently, broad-spectrum metalloproteinase inhibitors such as batimastat and marimastat, frequently employed to counter animal toxin MMPs, have exhibited diverse toxic side effects, including musculoskeletal toxicity, during clinical trials [20]. These factors present a formidable challenge in the introduction of conclusive alternatives to the current therapeutic agents.

Researchers have been interested in the utilization of traditional plant-derived drugs for treating animal toxin poisoning due to their widespread availability and broad-spectrum toxin-inhibiting properties. Hydroxybenzoic acids, a prominent subclass of phenolic compounds derived from plants, exhibit crucial biological properties, such as antioxidants, anti-inflammatory agents, antivirals, antibacterials, and anticancer agents. These compounds frequently serve as vital constituents or precursors in biologically significant drugs [21]. Notably, protocatechuic acid (PCA) (3,4-dihydroxybenzoic acid) and gentisic acid (DHB) (2,5-dihydroxybenzoic acid) are frequently present in citrus fruits (Citrus spp.) and members of the Allium spp. family, including onions [22,23]. PCA exerts anti-inflammatory effects by diminishing the production of inflammatory mediators, including IL-6, and by suppressing the expression of inflammatory genes and proteins through pathways involving NF-κB, MAPKs, and STAT 3. This action facilitates the amelioration of inflammatory disorders, including inflammatory skin diseases and osteoarthritis [24,25]. Additionally, PCA exhibits anti-allergic effects by inhibiting asthma airway remodeling, which involves suppressing the expression of type I collagen, fibronectin, and the deposition of extracellular matrix proteins [26]. Furthermore, DHB possesses anti-inflammatory properties by impeding the formation of lipopolysaccharide-induced PGE_2_ in the macrophage cell line RAW 264.7 through the inhibition of COX-2-derived prostaglandin-like compound synthesis. This compound has been employed in the management of rheumatoid arthritis. Moreover, DHB exhibits skin-lightening activity by impeding melanin synthesis through the reduction of tyrosinase synthesis or activity [27,28]. Furthermore, due to the fact that flavonoids derived from comparable plant sources and possessing monomeric structures similar to phenolic acids are frequently employed to inhibit the enzymatic constituents of animal toxins, including snake venom metalloproteinases, PCA and DHB hold potential as therapeutic agents for mitigating the inflammatory response induced by jellyfish stings. This study aimed to assess the inhibitory effects of PCA and DHB on NnNV toxicity, along with examining the efficacy of treatment for *N. nomurai* jellyfish sting wounds. In this context, the assessment encompassed the analysis of inflammatory factors, IL-6 and TNF-α, alongside inflammation-associated pathway proteins via cell stimulation experiments employing distinct toxin premixes. Furthermore, employing the “jellyfish sting-induced skin necrosis” model, this study encompassed histological and analysis of inflammatory regulation in NnNV-induced localized necrotic wounds. The capacity of PCA and DHB to impede enzyme components within NnNV was identified as a contributory mechanism to sting treatment. The findings of this study will establish a theoretical foundation for employing PCA and DHB as potential therapeutic agents for jellyfish stings, thereby addressing the existing limitations in the treatment options for local jellyfish sting symptoms.

## 2. Results

### 2.1. Suppression of NnNV Toxicity and Inflammatory Potential on HaCaT Cells by PCA and DHB

**Figure 1:**
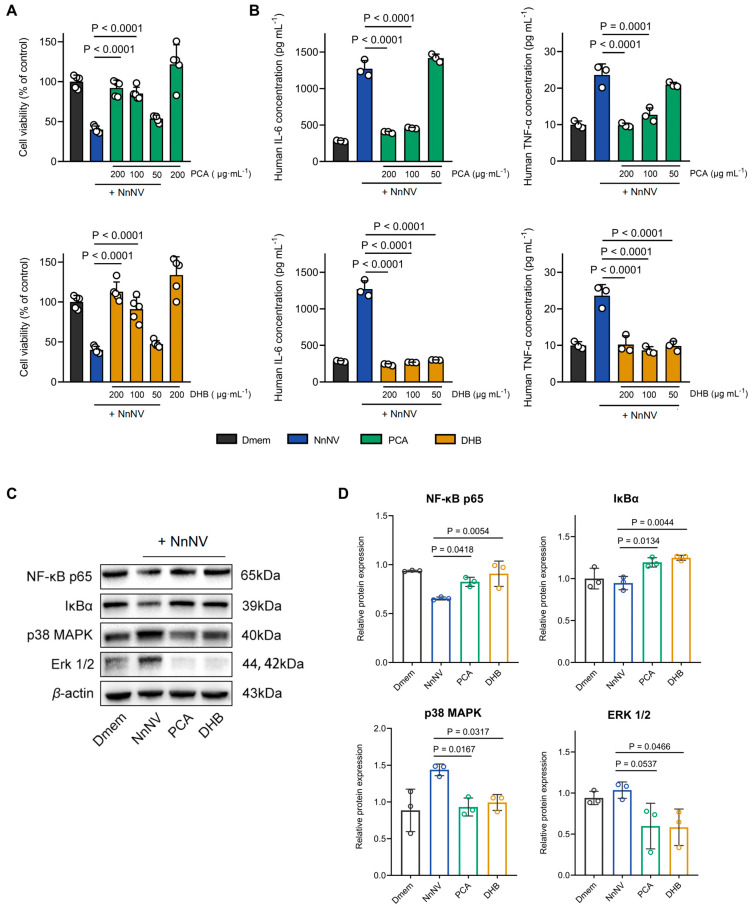
Inhibition of NnNV cytotoxicity and modulation of inflammation by PCA or DHB. (**A**) Cell viability was measured by treating HaCaT cells with PCA (green) or DHB (yellow) after 20 min of NnNV incubation (10 μg/mL). The highest experimental concentration of PCA or DHB was used as a control (*n* = 5). (**B**) Release of human IL-6 and TNF-α was evaluated after NnNV treatment (blue) and co-treatment with PCA (green) or DHB (yellow) (*n* = 3). Blank groups included untreated cells (Dmem, black) and the negative control group was treated with NnNV only (NnNV, blue). (**C**,**D**) Western blot of HaCaT cells after the above treatment. The protein levels of NF-κB p65, IκBα, p38 MAPK, and ERK 1/2 (*n* = 3). Data are mean  ±  s.e.m. *n* =  biological replicates.

A previous study [16] reported that NnNV contains a variety of enzymatic toxins that induce severe dermatitis or skin tissue necrosis upon jellyfish stings. The experiments confirmed that NnNV induces an inflammatory response in skin-associated cell lines, resulting in the up-regulation of inflammatory factors and subsequent reduction in cell viability. In this study, we treated HaCaT cells with varying concentrations of NnNV and determined an LC_50_ value of 11.91 μg/mL for NnNV based on cell survival (Figure S1). To investigate the potential of PCA and DHB in mitigating NnNV-induced skin toxicity, we compared changes in cell viability following treatment with different concentrations of PCA or DHB after a 20 min incubation with NnNV (Figure 1A). Concentrations of 100 and 200 μg/mL of PCA or DHB effectively inhibited NnNV cytotoxicity, restoring cell viability to levels similar to those observed with treatment using DMEM complete medium alone. Neither PCA nor DHB demonstrated the ability to neutralize toxin toxicity at a reduced concentration of 50 μg/mL. Additionally, when cells were treated with the highest concentration (200 μg/mL) in the drug control group, PCA did not demonstrate cytotoxicity nor an increase in cell viability compared to the blank group (*p* = 0.080). DHB did not exhibit cytotoxicity, but showed a slight increase in cell viability (*p* = 0.004). This suggests that in the phenomenon of DHB mitigating the cytotoxicity of NnNV, DHB’s ability to promote cell proliferation plays a minor role. Consequently, we conclude that both PCA and DHB can completely inhibit the cytotoxicity of NnNV on HaCaT cells.

The levels of human IL-6 and human TNF-α in cell culture supernatants were quantified using ELISA assays after treating HaCaT cells with NnNV alone or NnNV mixed with PCA or DHB (Figure 1B). The cells were treated with NnNV or the compounds for 24 h. NnNV stimulation resulted in a significant elevation of IL-6 and TNF-α concentrations in the cell culture medium, indicating the pro-inflammatory effect of NnNV on HaCaT cells and its ability to induce the release of inflammatory factors. The co-incubation of NnNV with 100 or 200 μg/mL of PCA led to a significant reduction in IL-6 and TNF-α concentrations in the medium, whereas DHB achieved this effect within the concentration range of 50–200 μg/mL. These findings suggest that PCA or DHB can effectively inhibit the release of NnNV-induced inflammatory factors from HaCaT cells, and co-incubation attenuates the pro-inflammatory capacity of NnNV, with DHB exhibiting a lower effective concentration. It is noteworthy that DHB at a concentration of 50 μg/mL can inhibit the release of inflammatory factors induced by NnNV, but it does not inhibit the cytotoxicity of NnNV. This indicates that further evidence is required to validate the potential connection between the inhibition of NnNV-induced inflammation and cytotoxicity by the compound. This inhibition may potentially reduce the damage to skin tissues or cells caused by NnNV through attenuation of the local inflammatory response, similar to the findings in toxin studies of other jellyfish species in Scyphozoa [29].

In pursuit of a deeper comprehension of PCA and DHB’s protective impact on cells under toxic influence, alterations in protein levels within the inflammation-linked MAP kinase and NF-ĸB signaling pathway were investigated (Figure 1C). Upon NnNV stimulation, the expression of NF-ĸB p65 displayed marked down-regulation (*p* = 0.0028), while p38 MAPK exhibited notable up-regulation (*p* = 0.0106) in comparison to the blank group (Figure 1D). Conversely, under the protective influence of PCA and DHB, these two proteins were reinstated to expression levels closely approximating those of the blank group. Simultaneously, relative to NnNV-stimulated cells, the intervention of both compounds elicited elevated expression of IĸBα and diminished expression of ERK 1/2. This observation suggests that the safeguarding effect of PCA and DHB on HaCaT cells involves the participation of the NF-ĸB and MAP kinase signaling pathways. However, it is worth noting that the trajectories of activation within these two pathways might diverge.

### 2.2. PCA and DHB Attenuate the Degree of Skin Necrosis Induced by NnNV In Vivo

**Figure 2:**
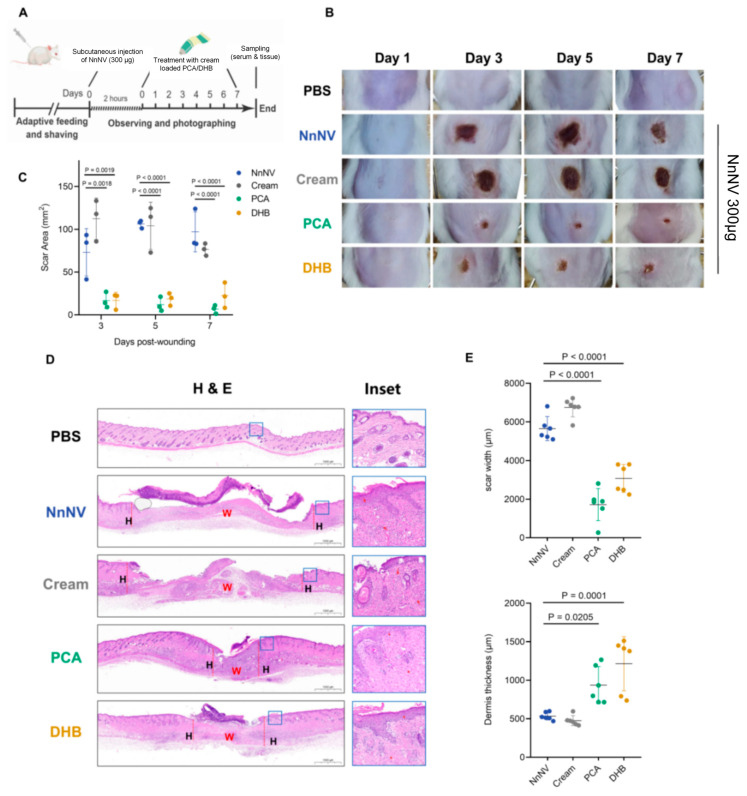
The topical application of creams containing PCA and DHB effectively mitigated NnNV-induced skin necrosis. (**A**) Schematic diagram illustrating the experimental setup for topical application of PCA and DHB to treat skin necrosis. (**B**) Representative images depicting the progression of skin necrosis on the backs of mice in each group on days 1, 3, 5, and 7 after subcutaneous injection of 60 μL of 5 mg/mL NnNV and continuous treatment with PCA (green) and DHB (yellow). (**C**) The necrotic area on the backs of each group of mice was assessed on days 3, 5, and 7 after NnNV injection and treatment (*n* = 3). (**D**,**E**) Histological analysis of Hematoxylin and Eosin staining was performed at the wound site in the dorsal skin of mice injected with NnNV and treated for 7 days. (**D**) Stain map illustrating the wound bed and surrounding healing tissue in the area of skin necrosis. H, healed skin tissue, shown on both sides of the red dotted line; W, wound bed, unhealed skin tissue, shown between the line. Scale bar = 1000 μm. The insets show enlargements of the blue box area. The red arrows indicate the presence of neutrophils. (**E**) Quantitative data on scale width and dermis thickness were obtained for each group (*n* = 6). The blank groups were injected with an equal volume of PBS buffer (PBS, black). The NnNV control group received only NnNV injection without the application of any cream (NnNV, blue), while the cream control group was injected with NnNV and applied cream without loading any drugs (cream, grey). Data are mean  ±  s.e.m. *n*  =  biological replicates.

The above results suggest that PCA and DHB possess the ability to neutralize NnNV toxicity on HaCaT cells. We further conducted experiments to validate the therapeutic efficacy of these compounds against NnNV-induced skin necrosis in vivo. To create the ‘Skin necrosis from jellyfish stings’ model, we subcutaneously injected 60 μL of 5 mg/mL NnNV into the dorsal region of mature male ICR mice. Topical treatment was administered using cream containing PCA or DHB for a duration of 7 days, during which the necrotic area and healing progress were assessed on days 1, 3, 5, and 7, respectively (Figure 2A). The NnNV control group received no treatment following toxin injection, while the cream control group received daily application of a blank cream after toxin injection. Comparative analysis with the PBS group (vehicle, 60 μL PBS buffer) revealed that mice in the NnNV group exhibited visible signs of localized skin necrosis on day 3 of the experiment, indicating the ability of jellyfish toxin at that specific dose to induce skin lesions in mice. The area of skin necrosis was significantly smaller in the PCA group (16.70 ± 5.33 mm^2^) and the DHB group (17.00 ± 5.53 mm^2^) compared to the two control groups (NnNV, 73.08 ± 16.00 mm^2^; cream, 112.31 ± 13.95 mm^2^) on day 3 of the treatment. Similar significant differences were also observed on days 5 and 7 of treatment (Figure 2B,C). Furthermore, no significant differences were observed between the two experimental groups treated with PCA or DHB at any time during the experiment. These findings indicate that both PCA and DHB effectively mitigated the skin toxicity of NnNV following injection, leading to a notable reduction in localized skin necrosis in mice induced by the sting model. Additionally, the therapeutic effects of both compounds were comparable. Additionally, H and E staining of the skin tissue at the necrotic area was conducted on the 7th day of treatment. The results demonstrated a reduction in the extent of necrosis in the entire skin cortex induced by NnNV, along with an improvement in the preservation of collagen fibers and a reduction in neutrophil recruitment, following the administration of PCA and DHB (Figure 2D). Quantitative analysis of scar width and dermis thickness within the necrotic areas revealed that the scar width in the PCA group (1718.80 ± 339.56 μm) and DHB group (3074.93 ± 296.48 μm) was significantly smaller than that in the two control groups (NnNV, 5651.70 ± 256.77 μm; cream, 6754.47 ± 200.12 μm). Moreover, the PCA group exhibited a narrower necrotic area compared to the DHB group (*p* = 0.0124) (Figure 2E). Additionally, the dermal thickness at the injury site was significantly higher in the PCA group (936.95 ± 98.41 μm) and DHB group (1214.14 ± 143.35 μm) compared to the two control groups (NnNV, 533.76 ± 20.28 μm; cream, 475.89 ± 26.24 μm). Notably, the histological analysis revealed no significant differences between the cream and NnNV groups, indicating that the blank cream utilized in the experiment lacked the ability to safeguard the skin against the damage inflicted by NnNV. In conclusion, transdermal treatment with PCA and DHB following NnNV injection significantly inhibited the development of necrotic skin lesions and neutrophil recruitment induced by the toxin, potentially facilitating the progression to the subsequent stage of wound healing.

### 2.3. PCA and DHB Facilitate Healing of NnNV-Induced Wounds

**Figure 3:**
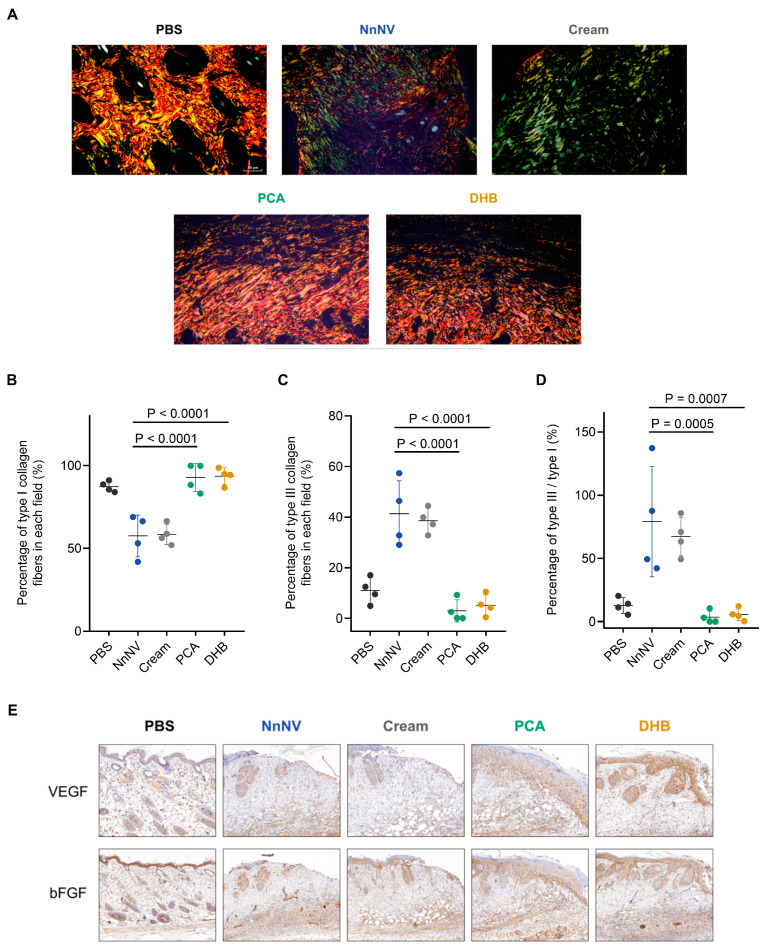
PCA and DHB enhance collagen remodeling and stimulate growth factor expression in NnNV-induced wounds. (**A**–**D**) Picrosirius red staining of the dorsal skin wound site in mice observed using polarized light microscopy after NnNV injection and 7-day treatment with PCA (green) or DHB (yellow) (*n* = 4). (**A**) Scale bar, 50 μm. (**B**) Quantitative data showing the percentage of type I collagen fibers in the wound bed field for each skin tissue group. (**C**) Quantitative data showing the percentage of type III collagen fibers in the wound bed field for each skin tissue group. (**D**) Proportion of type III/type I collagen fibers in the wound bed domain. (**E**) Immunohistochemical staining showing the expression of vascular endothelial growth factor (VEGF) and basic fibroblast growth factor (bFGF) at the margins of dorsal skin wounds in mice 7 days after NnNV injection and treatment with PCA (green) or DHB (yellow). Scale bar, 100 μm. Blank groups received injections of an equal volume of PBS buffer (PBS, black). The NnNV control group received only NnNV injection without cream application (NnNV, blue), while the Cream control group received NnNV injection with cream application but without drug loading (Cream, grey). Data are mean  ±  s.e.m. *n*  =  biological replicates.

Jellyfish stings frequently lead to delayed cutaneous allergic reactions, manifested by skin responses lasting several weeks. These reactions may include erythema, blistering, and epidermal necrosis. Persistent and severe adverse reactions can cause permanent skin marks, including keloids and color alterations [30]. Therefore, therapeutic interventions following jellyfish stings are crucial to facilitate wound healing. Furthermore, the diverse protease components found in jellyfish toxins degrade the dermal extracellular matrix (ECM) [31]. Collagen fiber, as a primary constituent of the ECM, plays a crucial role in providing structural support to resident cells during wound healing. Enhanced protection and remodeling of collagen fibers promote the healing of jellyfish envenomation of the skin. Thus, we conducted an additional investigation to assess the efficacy of PCA or DHB in promoting collagen remodeling in NnNV-induced wounds. Following 7 days of treatment, skin tissues were subjected to picrosirius red staining to identify the subtypes of collagen fibers (Figure 3A). Polarized light microscopy revealed distinct colors corresponding to different subtypes of collagen: type I collagen appeared as red or orange, while type III collagen and fibronectin exhibited a green hue. Type III collagen primarily participates in the initial phases of wound healing, gradually being substituted by type I collagen as the wound progresses into the remodeling stage [32].

The percentage of type I or type III collagen was quantified for each group using ImageJ based on the birefringence observed in the picrosirius red staining results. The NnNV control group received no treatment after toxin injection, while the cream control group was topically treated with a blank cream daily after toxin injection. The results demonstrated that the percentage of strongly birefringent red and yellow type I collagen in the skin tissue after PCA (92.83 ± 4.21%) or DHB (93.66 ± 2.53%) treatment closely resembled that of the PBS control group (87.32 ± 1.60%) and was significantly higher than the NnNV group (57.50 ± 6.29%) and cream group (58.34 ± 3.05%) (Figure 3B). Similarly, the percentage of weakly birefringent green type III collagen in the PCA group (3.00 ± 2.15%) and DHB group (5.15 ± 2.07%) showed no significant difference from the PBS group (11.01 ± 2.56%) and was significantly lower than the NnNV group (41.40 ± 6.50%) and the cream group (38.60 ± 2.45%) (Figure 3C). Additionally, the percentage of type III/type I collagen in the PCA group (3.44 ± 2.44%) and DHB group (5.69 ± 2.42%) closely resembled that in the PBS group (12.75 ± 3.11%) and was significantly lower than the NnNV group (79.11 ± 21.80%) and the cream group (67.32 ± 7.58%) (Figure 3D). These results indicate that treatment with PCA- or DHB-loaded creams led to a distribution of type I or type III collagen in the dermis that closely resembled that of normal skin. This effect may be attributed to the capacity of PCA and DHB to mitigate the detrimental impact of NnNV on the ECM at the onset of the experimental procedure, as well as the potential of PCA and DHB to enhance collagen fiber deposition and ECM remodeling.

Proteoglycan components, including heparin and heparan sulfate, within ECM serve as reservoirs for various growth factors, such as vascular endothelial growth factor (VEGF) and basic fibroblast growth factor (bFGF) [33]. During the wound healing process triggered by external injuries or diseases, the prompt remodeling of the ECM promotes the stable aggregation of growth factors. Moreover, VEGF and bFGF stimulate angiogenesis and tissue regeneration, playing crucial roles in promoting wound healing. To explore the protective effects of PCA and DHB on the ECM and their potential to enhance wound healing in NnNV-induced injuries, we assessed the expression of VEGF and bFGF at the wound edges using immunohistochemical staining (Figure 3E). The findings indicated substantial accumulation of both VEGF and bFGF adjacent to the epidermis in wound-edge tissues of the PCA and DHB groups, a distribution akin to that observed in normal skin tissues of the PBS group. Conversely, the NnNV and cream groups displayed minimal expression of VEGF and bFGF near the epidermis, with no observed gradient aggregation. Moreover, the overall expression of both growth factors was higher in the PCA and DHB treatment groups compared to the control groups, with the DHB group exhibiting higher expression. Based on these findings, we hypothesize that PCA and DHB safeguard the ECM by attenuating the toxicity of NnNV and promoting its remodeling. This process contributes to the re-establishment of a stable gradient of growth factors, including VEGF and bFGF, bound to the ECM. Consequently, the healing duration of jellyfish sting-induced skin necrosis is reduced. Overall, our findings suggest that PCA and DHB promote wound tissue remodeling towards a lower type III/type I collagen fiber ratio and enhance the expression of VEGF and bFGF, contributing to NnNV-induced wound healing.

### 2.4. In Vivo Inflammatory Modulation by PCA and DHB in Response to NnNV-Induced Impact

**Figure 4:**
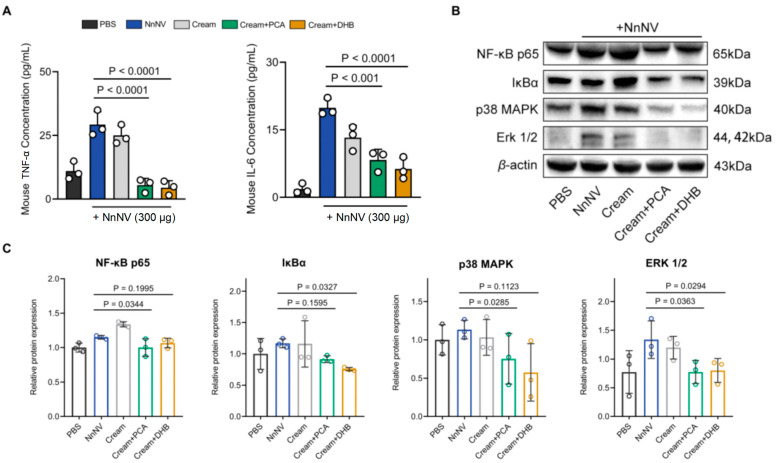
Changes in inflammatory factors and inflammation-related signaling pathway proteins in poisoned mice under PCA or DHB treatment. (**A**) Levels of IL-6 and TNF-α in mice after treatment with blank cream (gray), PCA (green), and DHB (yellow), respectively (*n* = 3). Blank groups received injections of an equal volume of PBS buffer (PBS, black), while the NnNV control group received only NnNV injection without cream application (NnNV, blue). (**B**,**C**) Western blot of skin tissue around the subcutaneous injection site in mice after the above treatment. The protein levels of NF-κB p65, IκBα, p38 MAPK and ERK 1/2 (*n* = 3). Data are mean  ±  s.e.m. *n*  =  biological replicates.

While the precise mechanism underpinning the toxic effects of *N. nomurai* venom remains incompletely elucidated, NnNV’s toxicity has been demonstrated to elicit dose-dependent systemic and local inflammatory responses across a spectrum of vertebrate models [34]. Subsequently, we undertook a comprehensive analysis of serum inflammatory factor levels and protein expressions pertinent to inflammation-associated signaling pathways within individual cohorts of mice in the aforementioned experimental paradigms. Notably, on the seventh day post-NnNV injection, IL-6 and TNF-α levels within the murine serum exhibited remarkable elevation in comparison to the control group administered with PBS buffer solely (Figure 4A). In contrast, mice subjected to a 7-day regimen of PCA or DHB treatment manifested significantly diminished IL-6 and TNF-α levels relative to the NnNV cohort. Application of the inert cream alone yielded no noteworthy reduction in inflammatory factor levels, thereby indicating that both PCA and DHB treatments jointly contribute to the amelioration of NnNV-induced systemic inflammation in mice.

Subsequently, we scrutinized the protein expression levels within two inflammation-associated signaling pathways, namely MAP kinase and NF-ĸB, across skin necrotic tissues (Figure 4B,C). Western blot analysis revealed that following NnNV injection, the expression levels of NF-ĸB p65 (*p* = 0.0339) and ERK 1/2 (*p* = 0.0289) within wound skin tissues exhibited a marked increase compared to those in the normal skin tissues of the control group. Persistent administration of PCA and DHB yielded diminished expression levels of NF-ĸB p65, IĸBα, p38 MAPK, and ERK 1/2 relative to the NnNV group. The expression levels of each protein within the cream group exhibited no discernible deviation from those of the NnNV group. Collectively, these findings imply the involvement of NF-ĸB and MAP kinase signaling pathways in the genesis of NnNV-triggered localized skin inflammation in mice, as well as in the mitigation exerted by PCA and DHB treatment. The amelioration of local skin inflammation facilitated by PCA and DHB contributes to the reduction in circulating blood levels of inflammatory factors and fosters the progression of wound healing processes at necrotic sites towards subsequent stages. This serves to reinforce the deduction that PCA and DHB indeed facilitate the healing of NnNV-induced skin wounds in the aforementioned murine experiments.

### 2.5. Inhibition Analysis of Enzyme Activity in Venom by PCA and DHB

**Figure 5:**
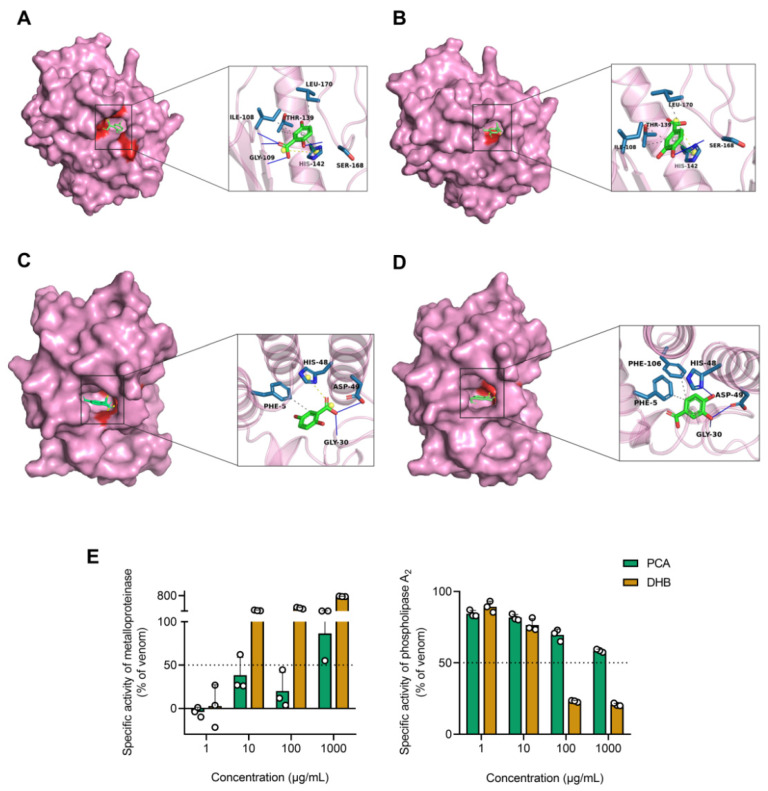
Molecular docking studies of PCA and DHB against snake venom metalloproteinase (SVMP) and cadmium-binding acidic phospholipase A_2_ (SVPLA_2_) with validation of in vitro enzyme activity inhibition. (**A**–**D**) Molecular docking complexes of two hydroxybenzoic acids with enzyme components, along with magnified views of the corresponding ligand–receptor interactions. The labeled amino acid residues interacting with the compounds are shown in blue. Specific ligand–receptor interactions are indicated by thin line segments, representing hydrophobic interactions (gray), hydrogen bonds (blue-violet), π-π stacking (green), and salt bridges (yellow). (**A**) DHB-SVMP complex. (**B**) PCA-SVMP complex. (**C**) DHB-SVPLA_2_ complex. (**D**) PCA-SVPLA_2_ complex. (**E**) Colorimetric determination of in vitro inhibition of MMPs and PLA_2_ activity in NnNV by PCA (green) and DHB (yellow), using compound concentrations ranging from 1 μg/mL to 1 mg/mL (lowest to highest dose tested). The results are expressed as the percentage ratio of each group to the NnNV-only control, data are mean ± s.e.m (*n* = 3).

The enzymatic components responsible for local damage caused by jellyfish venom have not been thoroughly characterized thus far due to challenges in availability, instability, and the complex synergistic effects of the venom [35]. Furthermore, purification of a single enzyme component and resolution of its structure have not been achieved [36]. The relevant proteomics studies had revealed that enzyme components, such as MMPs and PLA_2_ in NnNV, can be identified in snake venom as similar components with close homology [12]. To investigate whether the inhibition of NnNV toxicity by two hydroxybenzoids is caused by the inhibition of their primary enzyme components, molecular docking was conducted using individual enzyme fractions obtained from well-defined and documented snake venoms. The enzymes were snake venom metalloprotease BaP1 (SVMP) from *Bothrops asper* and snake venom cadmium-binding acidic phospholipase A_2_ (SVPLA_2_) from *Gloydius halys*, respectively. The molecular docking results showing docking scores and ligand–receptor interactions are shown in Table 1. The binding of PCA and DHB to the active sites of SVMP and SVPLA_2_ was observed, and their interactions with the two enzymes exhibited similarities. SVMP demonstrated favorable docking scores of −5.3 kcal/mol and −5.6 kcal/mol for DHB (Figure 5A) and PCA (Figure 5B), respectively. PCA exclusively formed hydrogen bonding interactions with SER168 amino acid residues, whereas DHB formed hydrogen bonds with SER168, GLY109, and ILE108 residues. Furthermore, both compounds exhibited similar π-π stacking and salt-bridging interactions with HIS142 amino acid residues, as well as hydrophobic interactions with ILE108, THR139, and LEU170 residues. SVPLA_2_ obtained docking scores of −5.4 kcal/mol and −5.5 kcal/mol for DHB (Figure 5C) and PCA (Figure 5D), respectively. Both compounds primarily exhibited comparable hydrogen bonding interactions with GLY30 and ASP49 amino acid residues, as well as similar hydrophobic interactions with PHE5 and PHE106 residues. Importantly, DHB formed salt bridge interactions with HIS48 amino acid residues, whereas PCA did not.

To validate the molecular docking outcomes of PCA and DHB concerning the two primary enzyme components of the toxin through in vitro experiments, we conducted additional investigations to determine the inhibitory potential of PCA and DHB on the activities of MMPs and PLA_2_ in NnNV using the colorimetric assay at drug concentrations 1000 times higher (ranging from 1 μg/mL to 1 mg/mL) (Figure 5E). The inhibition rate of MMPs activity was not displayed in a concentration-dependent manner due to the degradation of the substrate azocasein by PCA and DHB. Even at a concentration as low as 1 μg/mL, PCA and DHB completely inhibited the metalloprotease activity of NnNV (PCA, −4.21 ± 3.19%; DHB, 2.77 ± 14.05%). PCA and DHB exhibited concentration-dependent inhibition of PLA_2_ activity, demonstrating varying degrees of inhibitory potency within the 1000-fold drug concentration range. DHB (IC_50_ = 34.88 μg/mL) exhibited greater overall inhibition of PLA_2_ compared to PCA (IC_50_ = 5.15 mg/mL). Importantly, there was no noteworthy disparity in the inhibitory capacity between PCA and DHB at low drug concentrations (1 and 10 μg/mL). However, at high drug concentrations (100 μg/mL and 1 mg/mL), DHB exhibited significantly greater PLA_2_ inhibition than PCA (both *p* values < 0.0001). Based on the aforementioned experiments, we demonstrate that both PCA and DHB possess the capacity to inhibit MMPs and PLA_2_ in NnNV through both in vitro and silico. This ability may underlie the inhibitory effects of these two hydroxybenzoates on NnNV toxicity and their potential for treating local skin damage resulting from jellyfish stings.

## 3. Discussion

Jellyfish, belonging to the phylum Cnidaria, are free-swimming planktonic organisms. It is estimated that tens of thousands of jellyfish stings occur annually worldwide, particularly in tropical and subtropical coastal regions [37,38]. The growing jellyfish population in recent years, attributed to rising water temperatures and seawater eutrophication, has escalated the risk of jellyfish stings [4,39]. Antivenom is a common treatment following animal toxin poisoning. Besides the commercial IgG-type jellyfish antivenom, known as Commonwealth Serum Laboratories™ (CSL) box jellyfish antivenom, several other antivenom types are under development for various jellyfish species. Nevertheless, antivenom is ineffective against the localized symptoms primarily associated with jellyfish stings, and its limited cross-regional and cross-species efficacy, as well as its dependence on the clinical delivery environment, have constrained its development and application [40,41]. Conversely, small-molecule toxin inhibitors possess the advantage of broadly suppressing the key toxin components across various jellyfish species. Their ease of administration and efficacy make them the most promising avenue for treating jellyfish stings [7]. Phenolic acids, flavonoids and other polyphenolic compounds derived from plants find frequent application in the development of antagonists against animal toxins like snake, jellyfish, and scorpion venoms. Consequently, researchers have extensively investigated the inhibitory effects of these compounds on venom lethality and various enzymatic activities [19,42,43,44]. Flavonoids currently dominate the research on small-molecule compounds that counteract jellyfish toxin toxicity, while phenolic acid compounds have not been extensively investigated. Nevertheless, hydroxybenzoic acid compounds possess smaller molecular weights and exhibit easier storage and transport characteristics, rendering them more suitable for topical treatment of jellyfish stings [45,46]. In this study, we designated protocatechuic acid and gentianic acid as toxicity-neutralizing agents against NnNV and as agents for the treatment of jellyfish stings. Through modulation of cytokine release and proteins implicated in inflammatory pathways, protocatechuic acid and gentisic acid confer protection to skin cells against NnNV toxicity. The therapeutic effects of these two compounds were evaluated from various perspectives, including the inhibition of enzyme activity, collagen remodeling, total cortical restoration, modulation of growth factors, and inflammation. These compounds exhibit the capability to mitigate the prominent symptoms of dermatitis and local tissue necrosis associated with stings, which may contribute to the initial development of protocatechuic acid and gentianic acid as antidotes for *N. nomurai* jellyfish stings.

HaCaT cells are an aneuploid immortal keratinocyte cell line derived from adult human skin and find extensive application in skin biology research, including the investigation of skin disease mechanisms [47]. Following preincubation of NnNV with non-toxic concentrations of protocatechuic acid or gentianic acid, cell survival rates of 92.0 ± 4.4% and 113.0 ± 5.4% were observed, which are comparable or superior to the findings of previous studies utilizing small-molecule inhibitors like tetracycline, batimastat, and EGCG in the same experimental models [16,19,48]. Moreover, the levels of TNF-α and IL-6 in the medium were restored to those of the untreated control group, indicating that protocatechuic acid or gentianic acid can effectively deactivate both complex lethal and inflammatory proteins in NnNV. The antagonism of toxin toxicity by PCA or DHB is also shown not to be dependent on a particular component or pathway alone. This characteristic broadens their relevance as antidotes in a wider array of contexts, aligning with the perspectives of previous studies on inhibitor development [49].

While local injuries resulting from jellyfish stings exhibit certain common patterns, the clinical symptoms can vary significantly, influenced by factors such as individual body composition and the extent of exposure to jellyfish tentacles [50]. To explore the full potential of protocatechuic acid and gentianic acid in treating local injuries resulting from NnNV exposure, we administered a single injection of a high dose of jellyfish toxin protein (300 μg) to establish the ‘Skin necrosis from jellyfish stings’ model (Figure 2), which induced more pronounced symptoms of complete cortical damage compared to previous investigations [15,19,51]. Therefore, instead of employing the conventional histological analysis of local jellyfish sting injuries, we quantitatively assessed scar width and dermis thickness, commonly utilized in models of acute traumatic skin injury [52]. Our results visually demonstrate that the markedly reduced size of the initial skin injury within a three-day timeframe post NnNV injection, observed in both treatment cohorts as opposed to the NnNV control group and the cream control group. This implies that the topical application of protocatechuic acid and gentianic acid following toxin injection can effectively mitigate NnNV toxicity, indicating the potential for developing animal toxin inhibitors for external use as detoxifying agents in relevant domains [53,54,55].

In addition, skin remodeling takes place during the later phases of injury healing, predominantly characterized by collagen deposition and ECM remodeling [52,56,57,58]. Given that severe jellyfish stings often lead to the emergence of persistent symptoms like delayed skin allergic reactions and excessive scarring [5], we examined the distribution of type I and type III collagen fibers following intoxication (Figure 3), which mirrors the progression of the healing process [52,59]. The findings revealed a considerably accelerated healing process in the skin injury compared to the control group when subjected to continuous treatment with protocatechuic and gentianic acids, aligning with the observed pattern of VEGF and bFGF growth factor expression. Notably, type I collagen plays a crucial role in facilitating the adhesion and migration of keratin-forming cells, and its excessive synthesis and deposition are often associated with keloids and scleroderma. To ascertain the presence of any enduring symptoms that are challenging to alleviate in the final stage of treatment with protocatechuic and gentianic acids, and to enhance the assessment of the therapeutic efficacy, it is essential to extend the experiment to monitor and analyze the healing process of the jellyfish sting model over longer durations.

Jellyfish stings result in foreign body penetration and inflict cellular and cytoplasmic matrix damage via enzymatic components of the toxin. This prompts activation of the innate immune system, involving Toll-like receptors of immune cells that recognize pathogens. This leads to initiation of the MAP kinase, NF-κB, and IRF signal transduction pathways, thereby orchestrating inflammation through generation of inflammatory cytokines, chemokines, and antimicrobial peptides, culminating in activation and subsequent induction of an adaptive immune response [60]. Consequently, the current investigation scrutinized alterations in expression levels of NnNV-induced inflammatory factors and proteins within pertinent signaling pathways. This approach aids in comprehending the progression of jellyfish dermatitis and dissecting the mechanisms underpinning therapeutic agent efficacy. Notably, Western blot experiments conducted on HaCaT cells unveiled a notable reduction in the expression level of NF-κB p65 upon NnNV stimulation. This observation concurs with the findings from other earlier studies indicating NnNV’s dose-dependent suppression of NF-κB activation in HepG2 cells [61]. Noteworthy is the phenomenon that NF-κB signaling pathway activation exhibits contrasting trends at both cellular and organismal levels. This phenomenon might be attributed to the intricate nature of animal skin structure. Nevertheless, a comprehensive analysis of jellyfish dermatitis development mandates additional investigations concerning phosphorylated proteins within the signaling pathway, along with pivotal proteins both upstream and downstream.

Based on proteomic analysis, it was found that a substantial portion of NnNV’s enzymatic components, including metalloproteases and phospholipases, exhibit close homology to similar components in snake venom [12]. Consequently, we chose a well-characterized enzyme component from snake venom for molecular docking experiments with protocatechuic acid and gentianic acid. The selected enzyme, BaP1, is a P-I type zinc-dependent metalloprotease derived from the venom of *Bothrops asper*, a venomous snake species indigenous to Central America [62]. BaP1 demonstrates diverse activities associated with tissue damage, such as myonecrosis, skin necrosis, and edema. Additionally, an acidic phospholipase A_2_ that binds to cadmium, sourced from the venom of *Agkistrodon halys*, was also included [63]. Notably, protocatechuic acid and gentianic acid exhibited effective binding to the active sites of both enzymes. Docking results of baP1 with the matrix metalloproteinase inhibitor drug batimastat revealed similarities to our docking results in terms of hydrogen bonding, specifically involving amino acid residues SER168, GLY109, and ILE108 [17]. Furthermore, the hydrophobic interaction between protocatechuic acid and gentianic acid with BaP1 at residues ILE108 and LEU170 replaced the hydrogen bonding observed in the batimastat docking results. However, it is interesting that both protocatechuic acid and gentianic acid exhibited weaker binding energies to BaP1 compared to batimastat. The enzyme inhibition assays further validated the inhibitory effect of protocatechuic acid and gentianic acid on the activity of the key enzyme components in NnNV, thereby supporting previous studies that targeted metalloproteinases or phospholipase A_2_ for the treatment of *N. nomurai* jellyfish stings [42]. These findings align with the use of small-molecule enzyme inhibitors like marimastat, batimastat, and varespladib in the neutralization of diverse animal toxins [55,64]. An important consideration for the analysis of metalloproteinase and phospholipase A_2_ activities is that these two enzymes are prevalent in the venom of Cnidaria. Various biotechnological approaches have identified *Olindias sambaquiensis* and *Physalia physalis* in Hydrozoa, *Aurelia aurita* and *Cyanea capillata* in Scyphozoa, *Chironex fleckeri* and *Carukia barnesi* in Cubozoa, and *Actinia australis* and *Metridium senile* in Anthozoa, as species wherein metalloproteases or phospholipases A_2_ serve as major venom components [38,65]. Therefore, the remarkable inhibitory capability of protocatechuic acid and gentianic acid against both enzymes suggests their potential application in a broader context beyond *N. nomurai* to encompass other Cnidaria species.

## 4. Materials and Methods

### 4.1. Materials

Azocasein was purchased from Sigma Chemical Co. (St. Louis, MO, USA). 4-nitro-3-octanoyloxybenzoic acid (NOB) was purchased from Abcam (Shanghai, China). Protocatechuic acid (PCA), Gentisic acid (DHB), the BCA Protein Assay Kit, the Mouse IL-6 or TNF-α ELISA KIT, and the Human IL-6 or TNF-α ELISA KIT were purchased from Solarbio Science & Technology Co., Ltd. (Beijing, China). Sparkjade ECL super and Color enhanced Prestained Protein Ladder (10–180 kD) were purchased from Sparkjade Biotechnology Co., Ltd. (Jinan, China). NF-κB p65 (D14E12) XP^®^ Rabbit mAb (#8242), p44/42 MAPK (Erk1/2) (137F5) Rabbit mAb (#4695), IκBα (44D4) Rabbit mAb (#4812), p38 MAPK (D13E1) XP^®^ Rabbit mAb (#8690), β-actin (13E5) Rabbit mAb (#4970), and Anti-rabbit IgG HRP-linked Antibody (#7074) were purchased from Cell Signaling Technology, Inc. (Danvers, MA, USA). VEGFA Antibody (#AF5131) and FGF_2_ Antibody (#DF6038) were purchased from Affinity Biosciences (Changzhou, China). HaCaT cells were obtained from China Infrastructure of Cell Line Resource (Beijing, China). All other regents used were of analytical grade.

### 4.2. N. nomurai Nematocyst Venom (NnNV) Preparation

*N. nomurai* were collected from coastal waters near Qingdao, China in July of 2020. The method for extracting jellyfish nematocysts venom was as described in the past literature [13], but with slight modifications. Fresh jellyfish tentacles were cut and placed in seawater, and the tissue was gently stirred to autolyze. The tissue debris was removed by plankton net and the filtered liquid was collected. Centrifugation was performed for 15 min and the precipitate was collected to obtain the nematocysts to be broken. The nematocysts were resuspended by adding PBS buffer (pH 7.4) and broken by Ultraturrax (JY92-II, Scientz, Ningbo, China). A total of 90 cycles were performed, each cycle consisting of 30 s of homogenization treatment and 30 s of intermittent rest on ice. The extracts were combined and centrifuged at 15,000× *g* for 30 min at 4 °C. The supernatant was collected to obtain the toxin and split for freezing at −80 °C and labeled as NnNV. The toxin protein concentration was determined by the BCA method.

### 4.3. Enzyme Activity Determination

The inhibitory effects of PCA or DHB on the NnNV metalloproteinase and phospholipase A_2_ activities were determined by colorimetric assay as described previously [14]. Azocasein was used as a substrate for the evaluation of toxin metalloprotease activity, and the chromogenic substrate 4-nitro-3-octanoyloxybenzoic acid (NOB) was used for the evaluation of toxin phospholipase A_2_ activity. Currently, there is no universally acknowledged and specifically effective treatment for *Nemopilema nomurai* stings in the relevant research. To avoid inappropriate comparisons, we did not establish a positive control for our experiment.

Briefly, PCA or DHB was dissolved in PBS buffer (pH 7.4) and configured to a final concentration of 500 μg/mL, while NnNV was diluted with PBS buffer to a protein concentration of 1 mg/mL. PCA or DHB solution and NnNV were mixed in the same volume and incubated at 25 °C for 30 min, which was used as the incubation solution for performing the enzyme activity assay. The mixed incubation solution of PBS buffer and NnNV was used as a blank control, and the mixed incubation solution of PCA or DHB solution and PBS buffer was used as a negative control.

NnNV metalloproteinase activity was measured as follows. In total, 100 μL of 0.5% azocasein solution (*w*/*v*) and 25 μL of incubation solution of each group were pipetted into a centrifuge tube and incubated at 37 °C for 90 min. An amount of 200 μL of 5% trichloroacetic acid solution (*w*/*v*) was added to the reaction system to terminate the reaction. After centrifugation at 10,000 rpm for 20 min at 4 °C, 150 μL of supernatant was transferred to a 96-well plate and 150 μL of 2% NaOH solution (*w*/*v*) was added. The absorbance at 450 nm was measured by an Infinite M100 plate reader (Tecan Group Ltd., Männedorf, Switzerland), and the results of metalloprotease activity inhibition were expressed as a percentage (%) of each experimental group versus the blank control.

For the determination of NnNV phospholipase A_2_ activity, 25 μL of incubation solution of each group was pipetted to a 96-well plate, and 200 μL of PBS buffer was added to each well for dilution, followed by 25 μL of NOB (1 mg/mL in acetonitrile) per well for reaction. After the reaction system was placed at 37 °C for 60 min, the absorbance at 405 nm was measured, and the relative inhibitory activity of NnNV phospholipase A_2_ in each experimental group was expressed as a percentage.

### 4.4. Cell Culture and Cell Viability

HaCaT cells, an immortalized human keratinocyte cell line, find common application in the establishment of experimental models relevant to various skin diseases. HaCaT cells were cultured in DMEM (Hyclone Laboratories) containing 10% FBS (GIBCO BRL), 0.1 mg/mL streptomycin, and 100 U/mL penicillin G (Solarbio) at 37 °C and in a humidified atmosphere containing 95% air and 5% CO_2_. The systems were tested for the presence of Chlamydia and Mycoplasma contamination. Cell viability was determined by MTT colorimetric assay in the absence or presence of specified concentrations of NnNV and/or inhibitor as previously described [19]. In the study of the inhibitor’s ability to attenuate toxicity of the venom, different concentrations of PCA or DHB were mixed with NnNV (10 μg/mL) and incubated in 4 °C for 20 min to obtain the NnNV-inhibitor incubation solution, and then the cells were treated. Currently, there is no universally acknowledged and specifically effective treatment for Nemopilema nomurai stings in the relevant research. To avoid inappropriate comparisons, we did not establish a positive control for our experiment. The trypsin-digested cell suspension (5 × 10^4^) was inoculated in 96-well plates. After 24 h, NnNV or a different NnNV-inhibitor suspension was added and incubated for another 24 h. The medium was replaced with blank medium containing 0.5 mg/mL MTT. Four hours later, the medium was aspirated and DMSO was added, mixed in, and the absorbance values were measured at 490 nm. Cell viability was expressed by the ratio of absorbance values of the NnNV or inhibitor-treated sample group to the blank group.

### 4.5. Quantification of Inflammatory FACTORS levels by ELISA

Cell culture medium supernatant and mice serum were used as samples for ELISA experiments. HaCaT cells were inoculated in 6-well plates and treated with NnNV or inhibitors in the same steps as described above for cell viability experiments to obtain a model of NnNV-induced inflammation in HaCaT cells. Twenty-four hours later, the medium was transferred to sterile centrifuge tubes and centrifuged at 3000 rpm for 10 min at 4 °C, and the supernatant was aliquoted and stored at −80 °C. For the mice mentioned in the following methods, blood samples were collected via cardiac puncture immediately post-euthanasia. The mice plasma was naturally clotted at room temperature for about 30 min and centrifuged at 3000 rpm for 10 min at 4 °C. The supernatant was divided into separate centrifuge tubes and stored at −80 °C.

Cellular secretion levels of human or mice TNF-α and IL-6 were evaluated using the Human or Mice ELISA KITs, respectively, and experiments were performed according to the manufacturer’s instructions. For each ELISA experiment, we created a standard curve with standard substrates and calculated the concentrations of TNF-α and IL-6 in the supernatant using the standard curve for the current experiment.

### 4.6. Western Blot Analysis

Lysed cells or mice skin tissue were used for Western blot analysis. Treat HaCaT cells in 6-well plates with NnNV or inhibitors, and discard the old medium after 24 h. Cells were washed 3 times with pre-cooled PBS buffer (pH 7.4) and placed on ice. In total, 200 μL of Ripa High Efficiency Lysis Solution (G-Clone) containing PMSF and phosphatase inhibitor was added to each well and lysed on ice for 30 min with frequent shaking. The cell debris and lysate were scraped aside with a cell spatula and collected in a centrifuge tube. Following 10 min of centrifugation at 12000 rpm at 4 °C, the supernatant was removed and the sample protein concentration was determined by BCA Protein Assay Kit and the samples were diluted to the same concentration. The diluted samples were mixed with loading buffer and boiled at 98 °C for 10 min, divided, and subjected to SDS-PAGE. Mice skin tissue samples were washed with pre-cooled PBS buffer and placed in centrifuge tubes containing lysis buffer. The centrifuge tubes were placed on ice and homogenized with an electric tissue homogenizer for 3 s and then rested and cooled for 30 s. This was repeated until the samples were fully homogenized. The samples were centrifuged, diluted and mixed with loading buffer as described above, boiled, and dispensed for SDS-PAGE.

An amount of 15 μg of sample proteins was electrophoresed on 12% SDS-polyacrylamide gels, transferred to PVDF membranes and incubated overnight at 4 °C with specific primary antibodies (dilution ratio of 1:1000). After washing, the membranes were incubated with horseradish peroxidase (HRP)-conjugated secondary antibodies (dilution ratio of 1:5000) for 1 h at room temperature, and then the blotted membranes were visualized by enhanced chemiluminescence (Sparkjade ECL super). At least three Western blot analyses were performed for each target protein, and blot intensity was quantified by densitometry using ImageJ Fiji software (1.54i 3 March 2024).

### 4.7. Animal Maintenance

All mice in this study were 8-week-old male ICR mice, and all animal experiments were approved by the Ethics Committee of the Institute of Oceanology, Chinese Academy of Sciences, under animal experiment protocol number IOCAS/KLEMB/20170301. The mice were housed in a facility free of specific pathogens and at constant temperature and humidity, with 12 h of light and 12 h of dark, and a steady supply of food and water. The mice in each experimental group were housed together during the reproductive growth period and were randomly separated at the beginning of the experiment by a researcher who was unaware of the animals’ behavior and health status. Full attention was paid to whether conflicts between mice affected the health status of the epidermis.

### 4.8. Skin Necrosis from Jellyfish Sting Model and Preclinical Efficacy via Post-Treatment

The base cream prepared with cetostearyl alcohol, lauric acid, emulsifier, 1,2-Propanediol, and vaseline was heated to 60 °C in water, and 5% (*w*/*w*) PCA or DHB powder was added and stirred until there were no aggregated particles to obtain a mixed cream loaded with PCA or DHB. The mice were grouped (5 mice/group), shaved and depilated, and sedated for several days and observed for any adverse reactions on the back skin before the experiment. The ‘Skin necrosis from jellyfish stings’ model was established by subcutaneous injection of 60 μL of 5 mg/mL NnNV into the back of the mice, and the blank group was injected with 60 μL of PBS buffer (pH 7.4). The injection method of subcutaneous injection was used in order to simulate the process of puncturing the human epidermis by the tubules of jellyfish nematocyst during the occurrence of stings. After 2 h of delayed administration, apply 0.5 g of mixed cream loaded with PCA or DHB on the local necrotic area. The same quality of base cream without any inhibitor was applied to the cream control group, and the blank group and the NnNV control group were not treated with any cream. Standardized positive control drugs are not available. The same dose of cream was applied to the same area for 7 consecutive days, and the necrotic areas of the skin were photographed and recorded every other day. The mice were monitored for 8 days, with the humane endpoint of euthanasia, and collection of serum and skin tissue, performed as described above.

### 4.9. Histological Analysis of Necrotic Area Skin

The mouse skin tissue samples obtained in the above experiments were fixed in 4% paraformaldehyde (Biosharp, Hefei, China) for more than 24 h, and then the tissues were dehydrated and embedded. The tissues were sectioned along the wound centerline, Hematoxylin and Eosin (H and E) staining was performed, and the histological changes were observed by imaging using a vertical Leica microscope. ImageJ software was used to measure the thickness of the dermis, the width of the scar, and the number of hair follicles, and the average of 10 measurements in a fixed distance area was selected for quantification in each group of mice. To detect regeneration of damaged tissues, picrosirius red staining images were obtained by polarized light Leica microscopy, and the percentage of type I or type III collagen was measured.

### 4.10. Immunohistochemical Analysis

Mice skin tissues were fixed, embedded, and sectioned as described above, and heat-mediated antigen repair was performed using Tris/EDTA buffer (pH 9.0). Sections were then incubated with PBS buffer containing 3% goat serum for 1 h to reduce non-specific reactions. The sections were incubated with primary monoclonal antibodies VEGFA (1:1000) and FGF_2_ (1:1000) at 4 °C overnight. After rinsing, an appropriate amount of biotin-labeled goat anti-rabbit secondary antibody working solution was added dropwise, incubated at room temperature for 10 min, removed, and rinsed again. The HRP-labeled horseradish peroxidase labeling working solution was added dropwise and visualized using DAB Horseradish Peroxidase Color Development Kit (Beyotime, Shanghai, China) after incubation at 37 °C for 10 min.

### 4.11. Molecular Docking

Molecular docking was performed using Autodock Vina to predict interactions between proteins and small molecules [66]. The crystal structures of snake venom metalloprotease BaP1 (1ND1) and cadmium-binding acidic phospholipase A_2_ (1M8S) were retrieved from the RCSB Protein Data Bank (RCSB PDB, https://www.rcsb.org/, accessed on 7 January 2024) in PDB format. The molecular structures of protocatechuic acid and gentisic acid were obtained from PubChem (https://pubchem.ncbi.nlm.nih.gov/, accessed on 7 January 2024). The original ligands and water molecules were eliminated from the large molecules. Hydrogen atoms were added, and the small-molecule compound ligands were docked using the specified parameters of Autodock Vina. The top nine binding sites for each molecule were selected as outputs. The interactions between biological macromolecules and ligands were identified using PyMol software and PLIP web tool (https://plip-tool.biotec.tu-dresden.de/plip-web/plip/index/, accessed on 10 January 2024), and visualization files were generated [67].

### 4.12. Statistical Analysis

Sample sizes were determined on the basis of previous experiments using similar methods. Unless otherwise stated, all data points described are composed of three independent experiments taken from biological replicates of different samples and performed. The means and standard error of all data were obtained and one-way analysis of variance (ANOVA) corrected for multiple comparisons using Tukey’s statistical test with P adjustment setting <0.05 as the cutoff value. Graphpad Prism 7 was used to calculate statistics in bar graphs based on data using appropriate statistical tests, including one-way ANOVA, and multiple t-tests. Details of the statistical analysis were specified in figure legends.

## 5. Conclusions

In summary, the present study investigated the therapeutic potential of two hydroxybenzoic acid analogs, PCA and DHB, on post-sting skin affected by *N. nomurai* nematocyst venom. Our findings suggest that PCA and DHB effectively safeguard and rehabilitate regions afflicted by jellyfish dermatitis through multifaceted mechanisms, encompassing toxin toxicity neutralization, inflammatory milieu modulation, and promotion of collagen remodeling. This provides a conceptual foundation for expanding the repertoire of pharmaceutical agents catering to sting treatment and exploring the mechanistic underpinnings of localized reactions consequent to envenomation.

## Figures and Tables

**Table 1:** Residue interactions and docking scores of protocatechuic acid and gentisic acid against metalloproteinase and phospholipase A_2_.

Venom (PDB ID)	Compound Name	Autodock Score (kcal/mol)	Ligand–Receptor Interactions
Snake Venom Metalloproteinase, BaP1 (1ND1)	Protocatechuic Acid	−5.6	Hydrophobic Interactions (ILE108, THR139, LEU170), Hydrogen Bond (SER168), π-π Stacking (HIS142), Salt Bridge (HIS142)
	Gentisic Acid	−5.3	Hydrophobic Interactions (ILE108, THR139, LEU170), Hydrogen Bond (SER168, GLY109, ILE108), π-π Stacking (HIS142), Salt Bridge (HIS142)
Snake Venom Cadmium-binding Acidic Phospholipase A_2_ (1M8S)	Protocatechuic Acid	−5.5	Hydrophobic Interactions (PHE5, PHE106), Hydrogen Bond (GLY30, HIS48, ASP49)
	Gentisic Acid	−5.4	Hydrophobic Interactions (PHE5), Hydrogen Bond (GLY30, ASP49), Salt Bridge (HIS48)

## Data Availability

The authors will supply the relevant data in response to reasonable requests.

## Supplementary Materials

The following supporting information can be downloaded at: https://www.mdpi.com/article/10.3390/md22050205/s1, Figure S1: Cytotoxicity of NnNV at different concentrations on HaCaT cells. Determined an LC_50_ value of 11.91 μg/mL for NnNV based on cell survival.
