# Supplementary material for: Exploring the Efficacy of Hydroxybenzoic Acid Derivatives in Mitigating Jellyfish Toxin-Induced Skin Damage: Insights into Protective and Reparative Mechanisms
Source: Mar Drugs. 2024 Apr 29;22(5):205. doi: 10.3390/md22050205 (PMC11122885; doi:10.3390/md22050205)
Supplement: Supplementary file 1 [file marinedrugs-22-00205-s001.zip › marinedrugs-2977815-supplementary.pdf]

Supplementary Materials:

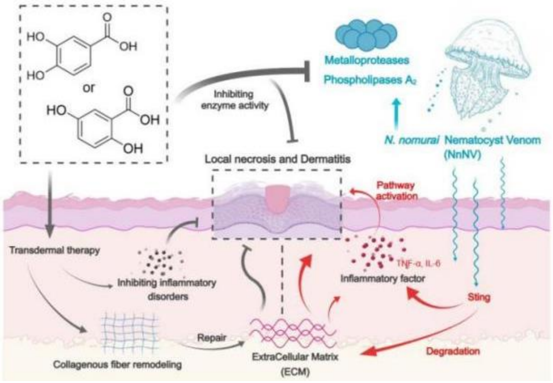

Graphical abstract

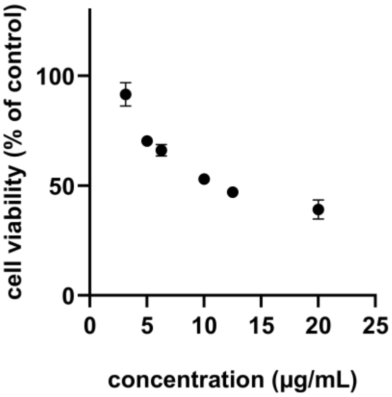

Figure S1. Cytotoxicity of NnNV at different concentrations on HaCaT cells. Determined an LC50 value of 11.91 μg/mL for NnNV based on cell survival. All results are representative of at least six independent experiments. Data are presented as mean ± s.e.m. (n=6).
